# Emulating sensation by bridging neuromorphic computing and multisensory integration

**DOI:** 10.1016/j.patter.2025.101238

**Published:** 2025-04-29

**Authors:** Antonio Bikić, Wolfram H.P. Pernice

**Affiliations:** 1 Department for Physics and Astronomy, Kirchhoff Institute for Physics, Heidelberg University, Baden-Württemberg, 69120 Heidelberg, Germany; 2 Department of Physics, CeNTech – Center for Nanotechnology, University of Münster, North Rhine-Westphalia, 48149 Münster, Germany

**Keywords:** artificial intelligence, touch, representation, neuromorphic computing, feature binding, haptic

## Abstract

Multisensory perception produces vast amounts of data requiring efficient processing. This paper focuses on the multisensory example of touch in biological and artificial systems. We integrate philosophical theories of multisensory perception with neuromorphic hardware and demonstrate how classical sensory integration concepts can enhance artificial sensory systems. This approach bridges theoretical neuroscience and computational applications using philosophical tools. We contrast human touch perception, involving feature binding, with artificial perception in neuromorphic computing, where such integration is absent. Two theoretical frameworks are of interest, feature binding and modalities as conventional kinds, in evaluating their relevance to artificial touch. Our findings suggest that a hardware-tailored adaptation of the conventional modalities approach accurately reflects artificial touch perception. Unlike human perception, artificial systems process sensory data separately, lacking binding mechanisms. We explore the implications of these differences, highlighting challenges in replicating human sensory experiences and the role of subjective experience in perception.

## The bigger picture

Can machines experience the world the way humans do? While biological systems, such as humans, naturally integrate, for instance, haptic sensory signals (such as pain, pressure, and vibration) into unified experiences that have meaning, artificial systems lack this ability. This integration, known as “feature binding,” seems to be a fundamental process in the brain that combines inputs from different senses into coherent perceptions of objects and environments. In contrast, artificial systems process sensory data in a modular, isolated fashion. This raises the question: do intelligent machines perceive the world in fundamentally different ways, and could this lead to different, but not necessarily worse, responses and functionalities?

As artificial intelligence systems become increasingly embedded in real-world environments, from robotics to prosthetics, understanding the nature and limits of machine perception is crucial. Neuromorphic hardware (engineered to mimic aspects of the human brain) offers a promising step toward more integrated and adaptive sensory processing. Yet, these systems still lack access to subjective experience, prompting us to ask whether information from conscious perception can be translated into hardware-readable formats—and if so, what information is essential for emulating sensation? Drawing from philosophy, neuroscience, and engineering, our perspective explores how differences in sensory integration shape different forms of intelligence. Clarifying these differences will be crucial for designing machines that can interact with the world in more natural, flexible, and robust ways.

## Teaser

What is the difference between artificial and biological perception? Bikić and Pernice explore the gap between biological and artificial sensory processing with a focus on multisensory integration in touch. They argue that artificial systems lack feature binding mechanisms, which makes them different from biological (human) perception. Their findings offer a theoretical framework for neuromorphic computing, with implications for AI-driven robotics, prosthetics, and sensory systems, paving the way for more adaptive and energy-efficient artificial intelligence.

## Introduction

Understanding how neural representations relate to one another in the brain can be illuminated by a classic thought experiment introduced by William Molyneux, now known as “Molyneux’s problem.” It asks: if a blind person, capable of distinguishing a cube from a sphere only by touch, were to gain sight, would this person be able to identify these objects correctly by sight alone, without touch? Current theories as well as experimental results suggest that this is not the case.^1^^,^^2^

Interestingly, Molyneux’s problem entails another, often overlooked, question, which we address in this paper: what do two neural representations of the same object have in common when they are represented by two different senses? Neural representations would in that case refer to the idea that the brain encodes perceptions, knowledge, and the like in its tissue. This means these encodings would *stand for*, for example, certain objects external to the brain, for instance, seeing a wolf and smelling one. If the latest experimental results prevail, neural representations by different senses do not have too much in common.

Although it is possible to repurpose some of the human senses to provide equivalent information if one modality is missing, it is limited due to, for instance, the spatial resolution of skin pressure receptors or photoreceptors in the eye. If we were to ascribe functions to organs, we could say that locating lines or dots in allocentric space is a function that can be performed by either the eye or the skin, depending on which sense is available.^3^

Thus, it seems impossible to semantically connect neural representations unless they are perceived simultaneously. While there are methods, like the “representational similarity analysis” (RSA), capable of comparing representations generated by the same or different senses,^4^^,^^5^ representations obtained by this method are already perceived simultaneously. Molyneux’s problem equally addresses current challenges in neuromorphic computing and artificial neural networks (ANNs) research in general. Integrating multiple sensory inputs still remains a fundamental barrier to the emulation of human-like perception in artificial systems.

Thus, if the perception of an object is given separately in different modalities, there seems to be no isomorphism between these objects, that is, no structure-preserving mapping. Unless *experienced* simultaneously, neural representations have no links that make interlocking possible. Experience, as we understand it, is the subjective process that incorporates emotions, reflections, and forms, as it were, a certain perspective on reality. It is a process for which we currently do not have a satisfactory theory that could explain the chasm between what we measure in the brain and what the living being experiencing something perceives (the “explanatory gap”). Additionally, if we take the neural tissue as the basis for neural representations, then representations bear no similarities to the objects they represent. The same is true for the parts of the hardware that we interpret as representations of data: the manipulated hardware of the memory device shares no resemblance with the object it represents.

Now, does the unexperienced artificial representation of touch lack a crucial property, or do these considerations have little to no meaning in practice? We suggest that the subjective experience of a neural representation, for which we do not yet have a theory with sufficient explanatory power, can contain more information than an unexperienced representation.

Nevertheless, subjective experience would not necessarily have to be labeled a different kind of substance or even “a-physical.” For a representation to work properly, it does not require similarity to other representations or aspects they describe. Experiences, however, can be similar to one another and can generate new information by referencing one another: songs can be similar to one another, while the neural representations of these songs do not have to be similar in structure. Moreover, experiences of representations can evoke references not contained in the representational data: wetness, for instance, is an experience made by humans without having hydrosenses.^6^

Regarding the somatosensory system, there is sufficient evidence for classifying touch as a multisensory perception,^7^^,^^8^^,^^9^^,^^10^ provided through several modalities. “Multisensory perception” is any cerebral process of integrating information from at least two different senses. The result is a comprehensive representation of the object in question. Take the surface texture, the temperature, and the consistency of an object and imagine these features experienced as three components of a single touch representation: does the process of multisensory integration produce a sensory perception—a subjective *experience*—that can provide more information about an object in its entirety than the separate processing of the modalities could have?

We review two approaches classifying touch, namely “(feature) binding” and “modalities as conventional kinds,” and show some of the recent development in neuromorphic touch systems. Feature binding refers to the process of integrating different stimuli, like acoustic and visual inputs, into coherent representations of an object, and it is essential for perception. The term “conventional kind” refers to objects that are artifacts. These need to be understood or rely on social conventions in order to exist as they were intended, like the concept of money. A conventional kind is the opposite of a “natural kind” (like a neutron star).

We suggest that, for neuromorphic devices, a multisensory approach with no feature binding describes *mutatis mutandis* more accurately how data are composed, processed, and memorized in neuromorphic devices. Based on that, we emphasize the dissimilarity of representations and experiences and formulate some challenges for the emulation of knowledge gained from experiences, in particular, if experiences allow for the accumulation of additional information not contained in the representation of a specific object. We define, for our purposes, the term “emulation” as an instantiation of every aspect of evolutively grown behavior that is substrate independent, can be modeled and governed by a program, and is useful for the construction of a device.

We chose neuromorphic hardware as a paradigm, since this kind of hardware implements carefully chosen structures of the brain that can be used to build a computer chip. This kind of hardware can be adapted specifically to the architecture neural computation needs and enables in-memory or parallel computation. Through this, neuromorphic hardware saves energy by orders of magnitude and speeds up the learning processes of artificially intelligent systems significantly. While still in the developmental stage, this kind of hardware makes systems with a reasonable energy consumption possible. It also paves the way for systems that can process enormous amounts of data locally, which in turn enables a functionality similar to the requirements for a living being that has to survive in a real-world environment.

## Neuromorphic touch sensors

Neuromorphic computing facilitates the creation of energy-efficient and rapid hardware overcoming the so-called “Von Neumann bottleneck,” where the central processing unit (processor) of a conventional computer processes the data faster than they can be taken from the memory. Since the processor has to wait, the performance is slowed down.^11^

This phenomenon is becoming a problem, since Moore’s law might come to an end, meaning that the transistors on a chip will stop doubling at the rate of about every 2 years, while the costs can be halved. Thus, Von Neumann computers can be criticized for their architecture continuously transferring data between memory and the processing unit. This process is very time consuming because the processor is forced to run idle for a period of time while waiting for the data. Additionally, Von Neumann computers are not designed to implement in-memory processing, meaning they are unable to process data in parallel. Orienting toward the way the brain processes data overcomes these two difficulties with parallel data processing and removes the need to store data in locations where they are not actually computed. The result is energy-efficient and fast hardware ideally suited to mobile and permanent use and data-intensive tasks without the need of a data center.

One of these tasks is processing the variety of behaviors performed by the somatosensory system, especially touch. In showcasing a few recent developments in neuromorphic touch systems, it becomes clear why technology, in order to be described as intelligent, needs a specific form of data processing.

Artificial skin, for instance, is capable of mimicking the sensory feedback and the mechanical properties of natural skin. Wang et al.^12^ presented a so-called e-skin that implements multimodal perception and can generate neuromorphic pulse-train signals while running on low operation voltage and low power consumption. While being monolithic in structure, stretchable, and at the same time soft, the e-skin is able to communicate pressure signals, allows for soft interactions with the surroundings, can identify temperature, and can encode these into stimuli in the form of electronic signals.

Neuromorphic hardware constitutes a deviation from ANNs based on multilayered perceptrons in that it can implement “spiking neural networks” (SNNs), meaning networks that integrate time into their artificial representations of information and transmit information only when a certain threshold is reached. These networks are fast and energy efficient and can be specifically designed to focus on reaching a goal with as few and as early spikes as possible.^13^ Birkoben et al.^14^ presented a piezoelectric sensor device capable of emulating tactile representations. The adaptable tactile sensor is based on an SNN. The sensor enables the encoding of mechanical quantity into spikes while adapting to the output frequency. Here, the environment becomes accessible in the tactile dimension, allowing the system to process information with a reasonable energy consumption and to convert mechanical to electric signals through a piezoelectric material. Neuromorphic tactile sensors can also perform edge orientation, for example, with the NeuroTac sensor.^15^ In combination with a camera, this neuromorphic sensor is capable of detecting edge orientation with the prospect of usage in prosthetics and robotics.

Categorization of different textures is another challenge for a reliable emulation of touch. However, Rongala et al.^16^ were able to present a neuromorphic system capable of categorizing 10 different textures, from glass to skin, with a 97% accuracy. The recognition and differentiation between different textures in humans is relevant because we can distinguish these textures even when finer than what is detectable via those senses with which we actually touch the texture. The texture’s spatial period seems to be translated into a corresponding spiking frequency. Mastella and Chicca^17^ have presented a neuromorphic system facilitating the detection of the range that inhabits the encoded frequency. This made the decoding of the texture’s spatial period and an implementation into integrated circuits possible, allowing for texture decoding. Prosthetics based on neuromorphic hardware encode, for instance, information from sharp objects to the person using the prosthetic. These prosthetics are also efficient tools for pain-detecting tasks.^18^ Also, touch can be restored after hand amputation with prostheses based on neuromorphic hardware, since these systems can emulate firing dynamics of mechanoreceptors in the skin to a certain degree.^19^

Besides the advantage of energy efficiency and speed, neuromorphic hardware—in contrast to ANNs running on conventional hardware—can actually benefit from neuroscientific insights^20^ and map neural computation functions onto the physical structure of a device.^21^ As seen here in the diverse touch-related examples, many neuromorphic devices can interact with the human body by means of electronic and electric signals. These devices can also use and detect patterns encoded by the human somatosensory system. The SNN approach paves the way to, eventually, integrate the links between the ion channels responsible for the transduction of force and the mechanoreceptors relevant to the translation of mechanical stimuli into electric potentials.^22^ Spiking in biological neural networks is closely related to ion channels, making the neuromorphic architecture perhaps even more suitable for exploiting this way of transmitting information.^23^

All of the exchanged signals between the devices and the organism are measurable values. The different modalities that these sensors include can be denoted by an observer as “touch related.” However, the argument we present in the section “modalities as conventional kinds approach” also applies: there is no psychological structure or causal instance underlying the touch-related modalities that all sensors in the artificially intelligent system would have in common. The hardware that we build is not geared to use the process of binding to create structures that can be experienced. Instead, as in the case with the piezoelectric material used to convert mechanical to electric signals, we measure the voltage to figure out the pressure without having to bind features that end up in a subjective experience of pressure. The modalities that we individuate are conventional (see “modalities as conventional kinds approach”) and serve our purposes to determine what the system is actually doing with a component. But these components are not intrinsically touch related; they, for instance, just measure the voltage. The difference between such a neuromorphic device and an organism capable of binding features is that at some point the organism binds features to create experiences, whereas machines bind features neither at the modality level nor at some conceptually higher level.

## Molyneux’s problem and the knowledge argument

**Figure 1:**
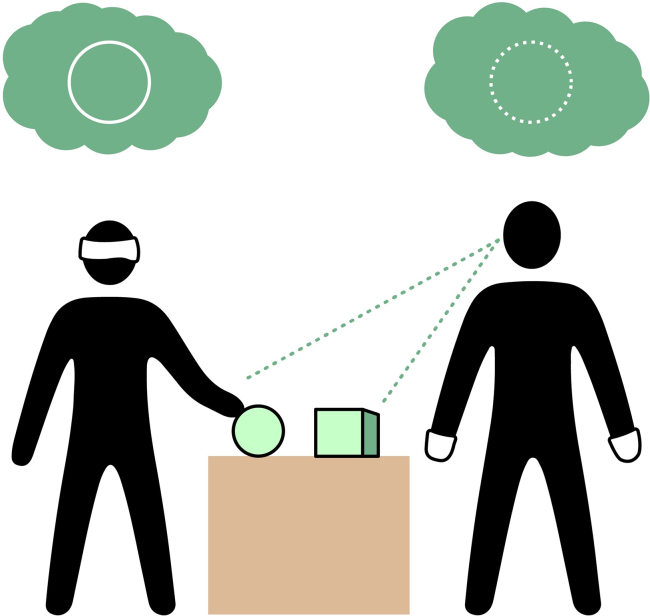
Molyneux’s problem If a blind person, capable of distinguishing a cube from a sphere only by touch, were to gain sight, would this person be able to identify these objects correctly by sight alone, without touch?

Molyneux’s problem^24^^,^^25^^,^^26^ poses the following question: is a formerly blind person, capable of telling a cube from a sphere haptically, able to distinguish between these two objects by correctly naming them as cube or sphere by sight and without touching them? This problem (Figure 1), originally formulated by William Molyneux in 1688, was inspired by John Locke’s distinction between ideas acquired by one and by multiple senses.^27^ For a long time, the discussion of this gedankenexperiment had little to no relevance for empirical science. This changed when William Cheselden was able to remove cataracts from congenitally blind persons in the early 18th century. Further research in the 19th century shed light on this topic through more experimental data and also influenced theories of vision suggesting that the vision system of newborns resembles that of a formerly blind person who gained sight.^28^

In the past decades, some reliable empirical data were gained when five children between the ages of 8 and 17 with very little to no eyesight were able to see after treatment. The patients were able to distinguish between objects visually but unable to establish a relationship between objects they knew from touch and those they were seeing now. After a few days, however, the patients were able to combine these two mappings across the two different modalities of perception.^1^^,^^2^ Thus, it seems likely that they were able to differentiate between the two objects not only in terms of knowing that their shapes were different. Rather, they were able to differentiate between these objects *as* cubes and spheres. Recent studies have also shown that infants with visual impairment are not able to combine auditory and tactile information the same way as their peers without impairment. This finding suggests that the experiences produced by the visual system especially seem to play a significant role in the modification of non-visual inputs.^29^

**Figure 2:**
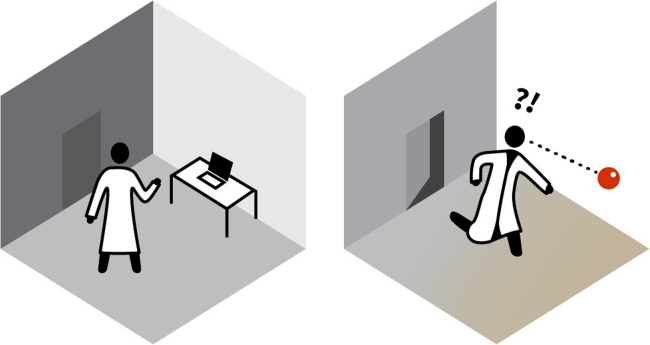
The knowledge argument Can physical knowledge account for all aspects of conscious experience?

It is worthwhile to juxtapose Molyneux’s problem with another influential thought experiment: the “knowledge argument.”^30^^,^^31^ Imagine a brilliant scientist named Mary (Figure 2) in a room where she is forced to study the world through a black and white monitor. After a while, she has acquired all of the information concerning human color vision but has never actually seen a color. When she finally gets a color monitor, sees a color, and experiences, for instance, the color red while seeing a ripe tomato, does she learn something new? If she had all of the physical information before and she learns something new after seeing the color red for the first time, then, as some might conclude, not all information is physical. This thought experiment was originally developed within the debates on phenomenal experience (qualia) and the question of *what it is like* to, for instance, see something red. In the same article by Frank Jackson,^30^ another example is perhaps even more instructive: imagine a person being able to see a color no other person can perceive. No matter how much information about the brain of the person is gained, with our current theories, it seems impossible to know what the phenomenal experience of this color is. This argument was designed as an objection to physicalism, but it also provides another valuable insight into the context of Molyneux’s problem: the simultaneously and subjectively experienced sum of neural representations provides (at least) a new quality of information without having to be a-physical.

In the context of the knowledge argument, the relevant addendum to this finding is that neural representations of the same (perceptual) phenomenon formed by different sensual modalities contain different aspects of said phenomenon but in another “format” than subjectively experienced representations. This means that neural representations of the same phenomenon are not related to one another, unless they are subjectively experienced together, the phenomenon of synesthesia in this case counting as an exception.

Looking at our experience, this comes at no surprise; otherwise, everything that is hard would have to smell the same and everything that is red would have to feel the same way. What happens in the case of different sensual modalities that form distinct neural representations is that they capture the same property in different ways, for instance: a cube is captured haptically and visually. Thus, something that is haptically round does not visually look that way but haptically feels that way. We normally learn these things simultaneously if our senses work properly, but the mere fact of a holographically projected sphere that is not haptically round shows that there is no necessary connection between the visual and the haptic properties.

Our current vocabulary usually refers to the concept of “representation” when we want to explain the basis of encoded information that is needed to act. The term “representation” is widely used in different disciplines, including philosophy (of mind), linguistics, cognitive science, neuroscience, psychology, and artificial intelligence (AI). Despite many considering it a central component of the mind, the meaning of representation is quite vague. Representations are deemed to be central components of the production of behavior of organisms. Cognitive science uses this term to refer to a basis of cognition, while neuroscience uses it to indicate “behaviorally relevant information” that can be found in cerebral structures like neurons or circuits. The latter is more of a correlational usage. Dynamic systems and connectionist approaches to cognition, on the other hand, often renounce the idea of a patent representation equipped with meaning and reference to some aspects of the world. Behavior is seen as an output that is generated based on input data. An arm, for instance, does not have to be represented in a central pattern generator to enable the system to reach for something. If the term “representation” is applied in such contexts, it can only refer to single (artificial or biological) cells that correlate with kinematic variables like position and speed.^32^

Representing the world in the form of neural representations and experiencing this world on the basis of these representations are two different ways of forming an epistemic access to the world, where the latter depends on (or supervenes) the former. Cross-modal mapping of different modes of perception is possible if the phenomenon is perceived simultaneously in these perceptual modes. In that way, the information describing an object haptically does not relate to the visual information of the object. If the empirical data hold, the idea that some visual features could dock onto some haptic features because they belong semantically together, like the converging edges of a cube forming a vertex and the haptic experience of something being sharp, is wrong. Rather, these two perceptual modalities are two (separate) ways of interacting with a perceptual phenomenon like a cube.

Take studying the neurophysiology of vision and representing gained insights with theories on the one side and experiencing objects captured by vision on the other: these are distinct ways of accessing the world epistemically. The perceptual phenomenon is not a platonic idea nor the phenomenal content of a cube. What is meant is the perceptual object (of a cube) being given to us in different ways.

## Touch as multisensory perception

Touch is believed to be one of the first senses to evolve^33^^,^^34^ and is, accordingly, a central form of perception. It is constituted by a plurality of modalities, which, for example, concern the quality of what is felt haptically, but can also include thermal awareness and altogether distinguishing between pleasant and unpleasant touch.^35^ These modalities are part of the somatosensory system producing additionally proprioception and the perception of pain. Take the sensation of heat and pressure: here, it is clear that these sensations are a result of different sensual modalities.

It is, however, unclear if and how to conceptualize the individuation of these different sensory modalities.^36^ Approaches theorizing about this individuation can be summed up by either suggesting a *binding* of the different modalities building up one sensual haptic modality or claiming these modalities remain *distinct*. We call these two approaches the feature binding approach and the modalities as conventional kinds approach. Contrasting these two approaches is useful, as it allows one to identify aspects of touch in living systems that can be used to make sense of the processes leading to artificial touch perception. The presented characterizations of artificial touch perception show two different ways of understanding the formation of perception.

### Feature binding approach

Binding as a concept tries to answer the neuroscientific question of how unified perceptions are generated from different sensory inputs. To date, it remains unknown how to comprehensively describe such multisensory integration, which is why binding still remains a problem and a relevant topic in neuroscience, since there is no theory that could describe this phenomenon sufficiently. It involves determining how neurons encode the stimuli of the external world, how these stimuli are in turn represented in the brain, and how neurons communicate with each other.^37^^,^^38^^,^^39^^,^^40^^,^^41^

Gaining deeper insight into these neural activities is essential for understanding the perceptual capture of an object, since the features of said object are processed in different brain areas. It remains unclear how the information of these different areas is integrated into coherent objects, including the binding of linguistic objects.^42^ Take acoustic or visual signals: due to their signal speeds they arrive at different times at the brain and cannot be processed at the same time and speed.^43^ It continues, therefore, to be quite remarkable (and puzzling) that the brain is capable of creating representations that integrate the information of different senses into coherent objects, establishing unisensory perceptual experiences.^44^

Haptic touch, for instance, is in itself multisensory but, according to the binding theories for haptic modalities, does not result in multisensory perception. These theories propose that we perceive distinct modalities such as pressure, heat, or grip as belonging to haptic touch and therefore to one another because we have a unisensory experience, but with different *already bound* components. Especially for touch, binding approaches^33^^,^^44^ suggest that the various modules of touch are related. Their structure and coordination toward unisensory perceptual experiences create a basis for a unified individuation of all haptic-touch-related modalities. These touch-related modalities of perception are first bound as a single modality and subsequently bound into coherent objects when combined with the processed signals of the other senses. This view is supported by conceptualizing coherence as a relevant criterion for binding meaningful multisensory information. In this approach, the correspondence in content (semantic congruency) serves as a significant factor for the binding of related multisensory information. The semantic content of a multisensory stimulus therefore plays a significant role.^45^ When, for instance, auditory and haptic signals are bound into coherent percepts in order to facilitate the recognition of a manipulated object, the brain is capable of binding the haptic and the auditory signals into coherent percepts based on their semantic congruency.^46^

It is known^47^ that binding of features occurs at both higher and lower association cortices. If we add this insight to the feature binding approach, we can formulate the following thesis: if it is true that binding occurs on both higher and lower association cortices for haptic and auditory inputs, it is reasonable to assume that haptic information is initially bound and afterward integrated with the auditory information. The reason for the primary binding of haptic information stems from the underlying semantic congruency of this kind of information.

The idea here is to bind touch-related features to coherent objects. According to this account, the different modalities of touch should be treated like properties of an object that the eye can predicate, such as shape, color, and size. According to this theory, touch predicates everything of an object that the different receptors can capture while still only capturing everything that is tangible.

### Modalities as conventional kinds approach

Another view would be seeing sensory modalities as distinct and, as a matter of fact, multisensory. Here, the different touch-related modalities bind features, but the modalities are in turn not bound into a unit. This is the approach of conventional kinds. Natural kinds—in opposition to conventional kinds—are entities that are not created by humans. Their functioning is independent of a proper interpretation, as would be needed in the case of money, for example. Conventional kinds, in contrast, are defined by humans and serve a clear purpose. In the case of senses, this means a rough but purpose-oriented distinction between the senses that is helpful in referring to the producers of percepts. This means either that there is no scientific basis for the senses in the conventional way or that, once we conceptualize senses as modalities, there are many more than claimed, namely—at least since Aristotle—five.^48^ According to this view, neither the causal origin of percepts, nor the objects or properties to which they refer, nor the resulting experiences provides a suitable basis for individuating the senses.^49^

Our experience of the world, then, is in general provided by much more distinct and distributed channels than we actually capture with our overreaching concepts of five senses. Just take touch as an example: there are at least 15 distinct modalities that we loosely subsume under the concept of touch.^50^ These include, but are not limited to, itch, pressure, hot and cold (temperature), pain, vibration, skin stretch (proprioceptive touch), perception of form/roughness, perception of texture, tickling, wetness, airflow sensation, electrical sensation, and grip control.^6^^,^^51^^,^^52^^,^^53^^,^^54^^,^^55^

According to the conventional kinds approach, the individual modalities are not interrelated. Modalities like weight, texture, and temperature are subsumed under touch, but this subsumption is not grounded in some other underlying psychological or causal instance. This approach is similar to the idea of informational channels or “avenues” that keep an organism up to date by providing information external to the nervous system. Since touch would rely on several of these informational channels, it would be a paradigmatic case of a multisensory perception. This is because stimuli activation profiles and other components differ significantly.^56^

Touch is believed to be one of the first senses to evolve, giving us the possibility of exploring our bodies and our sense of self,^33^ possibly also influencing the development of the “sense of agency,” a phenomenal experience (*quale*).^57^^,^^58^ The exploratory character of this sense is somewhat characteristic, as no other sensual organ interacts in such a direct way with the environment.^59^ If one were to assume a leading paradigm behind the touch-related modalities, it would clearly be the indexing of the surroundings through direct interaction. While this paradigm might be of explanatory value, it would inform about only the conditions for the different modalities to form features properly and would be ill equipped to provide a basis for a coherent system.

Human perception can be considered highly multisensory in the sense that what we feel and think about our experiences is modulated by other senses. This is, of course, if the percepts can be combined and are not impaired due to some dispositions as the ones cited in Molyneux’s problem. The case of affective touch is especially difficult, i.e., tactile sensation involving emotional and motivational facets.^60^ While interindividual touch can relieve negative affect and evoke pleasure, if other sensory cues indicate that the touching is unwanted, the same touch can be experienced as repulsive with no actual change in the signals transmitted by the person touching. In other words, internal states, neurochemistry, and the external context shape the hedonic value of touch.^61^ This sheer collection of different modalities, semantic values, and other factors, like a system of receptors dedicated to pleasant touch in hairy skin that is active when caressing touch is experienced,^62^ denotes a perceptual system that is too interrelated to be sharply individuated into scientifically distinguishable senses. Such description would suggest that the concept of touch relates to each one of these perceptual modalities like the term “inner organ” relates to the heart or the liver.

## Artificial perception

Considering the presented two theories regarding the formation of perception and having in mind that there is no actual feature binding in neuromorphic or even conventional hardware^63^ at any level, a reasonable way to conceptualize the way artificial representations are formed in neuromorphic hardware should be based on a “modified theory of modalities as conventional kinds.” Theories that involve binding when describing touch may be of value for the human experience of touch: we experience many of our direct explorations into the world as touch related. However, the information gained by neuromorphic systems stays separated and is not bound into coherent abstract symbols. Using binding theories to describe the way neuromorphic touch systems process information is, ultimately, inadequate. The claim that modalities are conventional kinds, on the other hand, provides valuable insights: the modalities, meaning the different sensors in machines, do not relate to one another because there is no underlying unity in these sensors. In fact, every artificial percept is multisensory but, in contrast to human experience, stays multisensory, since the integration of sensory information does not include binding in machines. This functionality is hardwired into the hardware of such machines.

These artificial percepts do not represent reality as objects completed with several features based on semantic congruency. Instead, the features stay separated and do not reveal more insight than the sum of the features contains or the features of different sensual modalities are added up to increase the perceptibility of an object.

The following examples of recent neuromorphic touch sensors illustrate the progress that has been made in terms of hardware. It also shows how a modified theory of modalities as conventional kinds can explain the epistemic access to reality established by neuromorphic hardware.

## The dissimilarity of representations and experiences

All of the features and information gained by the neuromorphic touch sensors that are stored as data and that are thought of as representations of some aspects of reality have no resemblance to the represented object. This means that the electronic or optical signals that make up the information gained from an object have neither qualitatively nor quantitatively anything in common with the represented object. For example, the number of photons per time unit can encode information but has intrinsically nothing to do with the apple it might represent. Additionally, the experiments conducted to answer Molyneux’s problem suggest that the modalities through which humans perceive the world provide different aspects of the objects perceived representationally. If an object is perceived visually, it does not mean that the haptic features can be connected to the visual “equivalents” if these two modalities are not perceived simultaneously. Moreover, not every aspect that can be comprehended visually can be comprehended haptically. For instance, colorfulness cannot be experienced haptically.

Humans are able to bind features from different senses into coherent objects. Identical to machines, the organic representations in the form of encodings through signals share no similarities with the actual experience made by humans when, for instance, seeing and touching a cube.

Visuohaptic representations may be functionally similar, but they are not connectable.^64^ If experienced simultaneously in an encompassing experience, the experiences of touching and seeing a cube, however, can actually be similar and provide points of reference. Assumed isomorphisms between, for instance, converging edges, represented visually and haptically, seem inexistent. Connections can, however, be made through simultaneous experience of all relevant modalities.

Gottfried Wilhelm Leibniz, for instance, suggested that shape representations generated by sight or by touch “isomorphically map onto the shapes being perceived, giving the tactile and visual representations a common structure, thereby providing a common feature for the once-blind to recognize the shapes.”^65^ Leibniz assumed some kind of an underlying geometry upon which reason could infer what features belong together if these objects have a *common structure*. Evidence for such common structure is still missing.

Imagine a scenario where the cognitive apparatus of a blind person represents the smell of a rose olfactorily. If this person is able to see the rose after treatment with four other flowers next to it, the person should in general not be able to connect the smell of a rose to the visual representation of the rose because of some underlying esthetics. In this case, there would be no common structure enabling such a connection.

As mentioned, the same is true for neuromorphic hardware: representations generated by different sensors are dissimilar to one another and have no resemblance to the object being represented. In contrast, human experiences *can be* similar to one another, can resemble the object in the outside world, and may influence the memory of an event or object.^66^^,^^67^^,^^68^ This relates to one of the insights from the knowledge argument: the physical information representing reality and the experience of reality are distinct from each other. This means that they are not assembled as different perceptual aspects of an object. Consequently, experiences *can* influence the storage or memory of information in humans.

Actually binding features of different sensory provenience into coherent objects requires simultaneity. Neuromorphic architectures, however, process data *in parallel* and not *simultaneously*, since simultaneity of events is established by a (conscious) observer. While humans can simultaneously perceive different sensory data mediated by different sensory organs, these sensory data converge into one experience, e.g., hugging a person. Binding of different features whose data arrived at the respective organs with time differences up to several powers of 10 into coherent objects is a key property of the human brain. The lack of feature binding is still one of the major problems for ANNs and SNNs when it comes to processing the world through symbols and poses a problem in terms of extrapolation.^63^

Every artificial percept is multisensory and stays multisensory, since the integration of sensory information does not include binding in machines. The features provided by the different sensors do not lead to the emergence of new objects (experiences) with new properties, such as magnetism in ferromagnetic materials like iron (individual atoms of iron do not have the property *magnetic*). Instead, the neuromorphic systems can use all of an object’s information in parallel. However, the current architecture lacks the combination of the features that would enable a symbolic view of the world.

The mentioned machines do not perceive the world subjectively, but they hold data that could be interpreted as a representation of the world. Representations, however, assemble parts of the reality without having to contain all of the information that can be experienced. Take the subjective experience of wetness^6^: although there are no hydrosenses in the human skin that could recognize this specific quality of a liquid, other modalities like thermal and mechanosensory features are used to determine if something is wet. Here, the data leading to a neural representation of the liquid does not contain information about the quality “wetness.” While it should, then, be impossible to subjectively experience this modality, a combination of the other data makes it possible to gain this information about the chemical compound.

This leads to the question of how to emulate the human perception apparatus without having to integrate the possibility of experience, qualia, and the like. The currently most promising idea is to create a version of a so-called *philosophical zombie*: an entity that is able to function the same way as a human behaves but with no conscious experience (see the next section).

Tan et al.^69^ constructed a system capable of multisensory cross-modal integration and recognition based on neuromorphic sensors using all of the five commonly known senses and tested it on simple tasks. Here, the spike number of different sensing modalities is, for instance, integrated to detect an approximating car faster using photomemristors that produce post-synaptic currents. Being a far cry from experiences of representations, this system produces a functionality very similar to human behavior for simple tasks. Features gained by different modalities are not bound but summed up in a way that produces a useful functionality previously identified as behavior in living beings.

More generally, conventional ANNs, such as convolutional neural networks (CNNs) and transformers, not only are not designed to implement the mechanisms of what neuroscience calls the binding problem, but this problem and the complex it forms are simply not relevant for the functioning of these systems. These architectures process information spatially and hierarchically (e.g., through hierarchical feature extraction), meaning they approach real-world problems different from biological systems. There are, of course, some alternative architectures that have drawn significant inspiration from neuroscience.

One such architecture is the aforementioned SNN, which processes information using spikes (discrete signals), enabling time-based feature encoding and dynamic feature association. However, training SNNs is significantly more complex than training conventional ANNs.^70^ One major challenge is the non-differentiability of spikes, which makes traditional gradient-based optimization methods ineffective.^71^ Renner et al.^72^ introduced a back-propagation algorithm for SNNs only recently. Additionally, current neuromorphic hardware, which is required for such computations, is not yet optimized for large-scale training, making real-time multimodal integration particularly challenging.

Another architecture leveraging neuromorphic computing is the oscillatory neural network (ONN).^73^ ONNs are designed to simulate neural oscillations, particularly gamma-band synchronization, which is of importance for the synchronization of rhythmic neuronal activity.^74^ One widely accepted theory of feature binding posits that synchronized neural oscillations mediate the integration of distributed sensory information.^37^^,^^75^ However, ONNs face significant challenges, including their sensitivity to noise, which disrupts the precise synchronization required for robust feature binding. Additionally, integrating ONNs into existing ANN frameworks remains a technical obstacle. Furthermore, despite these obvious advances made with those systems, both SNNs and ONNs still bind features very different from human cognition, highlighting the gap between current neuromorphic architectures and biological intelligence.

Emulating the process of extrapolation enabled by the experience of bound features is one of the main challenges in neuromorphic systems, sensing wetness being one of the easier tasks. Since experiences and representations are dissimilar, emulating the representations does not lead to the information gained when representations are also experienced.

## Philosophical zombies

**Figure 3:**
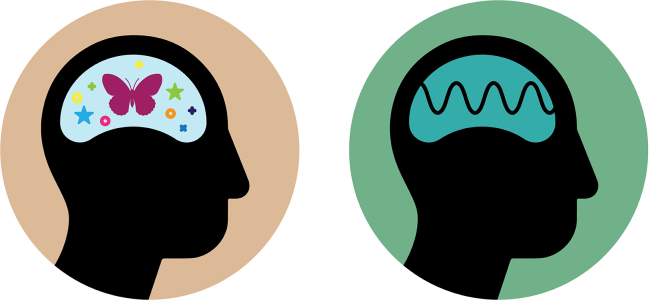
The philosophical zombie Left: human subject with phenomenal experience (qualia). Right: hypothetical entity without subjective experience but exhibiting identical behavior.

So-called “philosophical zombies” or “p-zombies” represent an influential conceptual tool for investigating the nature and possibility of consciousness and how it is situated in the physical world. Starting with the idea of automata, already discussed in Descartes, the last century systematized this concept further, developing gedankenexperiments governed by the notorious question: could there be creatures that are indistinguishable from humans but have no conscious experience? These entities can talk, exhibit pain gestures when exposed to violence, laugh, and so on—but with no mode of *what it is like to be* this particular p-zombie (Figure 3).

The p-zombie argument was often used to argue against the thesis that reality is fully reducible to the physical while rejecting immaterial substances at the same time (often called “physicalism”). It was developed further in a comprehensible and systematic manner by David Chalmers.^76^ Without having to assume that there are at the moment actual p-zombies, the mere fact that the existence of a p-zombie could logically not be impossible would suggest that a comprehensive physical and functional description of a system did not imply conscious experience.

Similar to the knowledge argument and for our purpose, our aim is not to debunk physicalism but to focus on a corollary of this thought experiment: is an artificially intelligent system able to exhibit functionality identical to human behavior without having conscious experiences? Or more precisely: is the totality of human behavior for an intelligent machine possible without conscious experience?

If an artificially intelligent system can function the same way a human can without having conscious experience, this particular property of a human could be neglected when constructing artificial systems. Meaning that engineers would never have to worry about building subjective experience into intelligent machines to improve or augment the functioning of the system. This would be possible because of the possibility that every action accompanied by conscious experience could be transposed into a functioning independent of conscious experience. Similar to the early conceptions of behaviorism, the subjective experience of an event can be neglected when generating the desired behavior and is therefore epiphenomenal.^77^^,^^78^^,^^79^

This thesis is backed by numerous recent artificial intelligence (AI) systems that obviously lack the property of conscious experience but exhibit a functionality that in many cases is very similar to human behavior accompanied by this very conscious experience. This is, among others, true for large language models,^80^ image classification,^81^ and also Turing-tested handshakes.^82^

The last is especially remarkable, since perception and sensorimotor tasks require much more computation than abstract thinking or reasoning, as captured in Moravec’s paradox.^83^

One could also ask more broadly: can connectionist artificial systems perform complex cognitive functions beyond basic behavior and pattern recognition without conscious experience? Higher-order cognition, such as abstract thinking and reasoning, can, to a remarkable extent, be emulated using ANNs or SNNs. However, some of these higher-order cognitive capacities are better realized through symbolic AI.

A defining characteristic of human thought is its metacognitive awareness and introspection. If these capacities are essential for emulating human behavior in the form of a p-zombie, then current AI systems lack crucial components, as they do not (or only minimally) incorporate these faculties. However, emerging research in neuromorphic architectures, self-supervised learning, and hybrid AI models could help in computationally approximating some aspects of self-awareness. Extending the p-zombie hypothesis to a broader range of human-like capacities would require an emulation of what we identify as the self. While the self is a complex and somewhat ambiguous concept, it broadly refers to the entity signified when humans use the personal pronoun “I”. Philosophical and biological research has provided detailed insight into this concept, which could be summarized in broad brushstrokes as follows: the sense of agency, i.e., the subjective experience of being in control of one’s actions.^57^^,^^58^ This sense of agency (a *quale*) and ownership helps determine which elements of one’s body and actions are perceived as “part of oneself.” If translated into engineering, such research could further develop the p-zombie hypothesis, particularly in AI systems seeking to mimic self-awareness and autonomous decision-making.

However, transitioning from a simulation of human behavior to a complete emulation (if such a thing is possible) presents numerous challenges. One of the most significant obstacles is that current AI systems lack understanding of common sense. While humans can also exhibit common sense deficits, AI’s shortcomings stem, among other factors, from the way it encodes and processes data. In ANNs, SNNs, and related architectures, situations are neither represented through abstract concepts nor grounded in the perceptual mechanisms of human cognition. This includes, for instance, Gestalt laws of grouping and feature binding, which are crucial to human perception and conceptualization.

From this perspective, it seems unlikely that AI systems and human cognition, given their fundamentally different approaches to encoding and processing information, will—in the current form—converge toward a shared structure of behavior or common-sense reasoning. Common sense encompasses implicit rules, general knowledge, high context sensitivity, and heuristic-based (intuitive) reasoning, all of which AI currently struggles to replicate. The last, in particular, poses serious challenges for ANNs, as demonstrated by issues such as shortcut learning^84^ and the Clever Hans problem.^85^

## Summary

Building on our main hypothesis, that experiences and representations are dissimilar, we established the thesis that emulating representations does not lead to the same amount of information that one gains when representations are experienced. The experience of wetness is one of the examples. Experiences of comprehensive and cohesive objects require, at least in humans, a process called feature binding. This process is deeply intertwined with the symbolic view of the world, as the Google Brain researchers^63^ suggested. This process is also important for extrapolation, a functionality that still needs to be developed further in intelligent systems.

Currently, there are two approaches for addressing this issue: either we should try to develop our intelligent machines further as they are and implement binding structures as suggested by Greff et al.^63^ or we should try to find ways to build systems that are at least a p-zombie in the sense that it does not need feature binding to exhibit the same functionality as a system that is capable of feature binding.

The example of touch and neuromorphic hardware lends itself to this explication, as touch is in itself multisensory and relates smoothly to Molyneux’s problem that (also) problematizes the similarities between neural representations.

Bare neural representations share no similarities with the facts they represent. They have no links via which they could interlock with representations that are different in kind, unless perceived simultaneously. Artificial representations in neuromorphic hardware can be added up in terms of salience, when acoustic and visual signals are converted into electronic ones. Adding up these signals can increase the salience of the signal indicating clearly that, for instance, an object is moving toward the system. These artificial representations, however, contain no binding of features and lack the creation of symbols that can be similar to one another because of their similar meaning. This claim, however, does not hold for artificial representations that are of the same kind: large language models, for instance, have mechanisms to determine whether a set of words is related. Thus, approaches not including feature binding describe neuromorphic hardware best.

Representations of different sensory organs of the same perceptual phenomenon contain different aspects of said phenomenon but are bound together afterward. Yet, the current paradigm of neuromorphic hardware offers no binding of these aspects into symbols and objects but rather keeps these features or parameters separated. In general, binding and symbols are considered strong criteria for robust extrapolation. And these structures are also important for experiences.

Experiences inform about the phenomenal aspects of colors and provide additional, derivative, information like wetness; but most importantly, however, in an experience, the correspondence in content serves as a significant factor for the binding of related multisensory information. Since binding problems also include the (subjective) unity of perception, the multisensory information is translated into *one* semantically congruent object.

The challenge lying ahead is the development of artificially intelligent systems capable of exhibiting functionalities that are comparable to information extracted from experiences. Neuromorphic hardware is a promising candidate for such emulations, since massive amounts of data can be computed locally with reasonable power consumption. Beside emulating the capability of sensing wetness and being able to react to colors without actually experiencing them, one major challenge for artificial touch perception is the production and detection of pleasant and unpleasant touch, since machines can measure quantitative but not directly qualitative aspects of touch. The realization of individually customizable pleasant touch would increase the acceptance of artificial agent-like devices, for instance, when determining how to plan a comfortable blood draw.

When it comes to the capacity of subjective experience, however, there still seems to be a lot to discover. There are structures in the neural tissue making sure that representations are treated in a very idiosyncratic manner that we call “subjective experience.” Whether finding these structures and ways to emulate them would increase the performance of neuromorphic devices is unclear, since current systems do no rely on human-like feature binding. It is therefore uncertain whether such structures would improve the machine’s functionality or turn out to be useless for our currently used hardware approach to controlled system activity.

## Acknowledgments

We thank the anonymous reviewers for their valuable feedback, which helped refine our arguments. We are also grateful to Stephan Sellmaier for his initial feedback on this project, to Nora Angleys for proofreading this article, and to Willem Rabe for assistance with the graphics. Finally, we thank CRC 1459: Intelligent Matter (10.13039/501100004869University of Münster, project no. 433682494) for partially supporting this research.

## Author contributions

Conceptualization, A.B. and W.P.; methodology, A.B.; investigation, A.B.; writing – original draft, A.B.; writing – review & editing, A.B.; funding acquisition, W.P.; resources, W.P.; supervision, W.P.

## Declaration of interests

The authors declare no competing interests.

## Declaration of generative AI and AI-assisted technologies in the writing process

During the preparation of this work, the authors used ChatGPT (OpenAI) and Gemini (Google) to supplement research engine queries for information on specific topics. After utilizing these tools, the authors carefully reviewed and edited the content as needed and take full responsibility for the final publication.
